# Chemical Surface Modification Methods of Resin Composite Repaired with Resin-Modified Glass-Ionomer Cement

**DOI:** 10.1055/s-0042-1755627

**Published:** 2022-10-11

**Authors:** Awiruth Klaisiri, Panupat Phumpatrakom, Niyom Thamrongananskul

**Affiliations:** 1 Division of Restorative Dentistry, Faculty of Dentistry, Thammasat University, Pathum Thani, Thailand; 2 Division of Endodontics, Faculty of Dentistry, Thammasat University, Pathum Thani, Thailand; 3 Department of Prosthodontics, Faculty of Dentistry, Chulalongkorn University, Bangkok, Thailand

**Keywords:** resin composite, resin-modified glass-ionomer cement, silane coupling agent, universal adhesive

## Abstract

**Objective** This study examined the chemical surface modification methods of resin composite repaired with resin-modified glass-ionomer cement (RMGIC).

**Materials and Methods** Ninety aged resin composite rods were produced and sorted into 9 groups of 10 specimens and surface modified with silane agent and/or bonding agent as follows: group 1, no surface modified; group 2, etch + single bond 2 (SB2); group 3, SB2; group 4, etch + RelyX ceramic primer (RXP) + SB2; group 5, RXP + SB2; group 6, etch + single bond universal (SU); group 7, SU; group 8, etch + RXP + SU; and group 9, RXP + SU. A clear silicone mold was placed on the top of specimen center, and then filled with RMGIC. The specimens' shear bond strengths (SBSs) were examined in mechanical testing equipment. To determine failure types, the fractured specimen surfaces were inspected using a stereomicroscope.

**Statistical Analysis** The data collected were analyzed using one-way analysis of variance, and significance level was operated using Tukey's test ( *p* < 0.05).

**Results** Group 8 had the greatest SBS, but it was statistically indistinguishable from groups 4, 5, and 9. The most frequent fracture mode was adhesive failure. High SBS was commonly associated with mixed failure.

**Conclusion** The use of bonding agents enhances the resin composite's wettability and allows it to bond to RMGIC. Moreover, the use of the silane coupling agent before applying bonding agent showed significantly higher bonding ability of resin composite and RMGIC interface.

## Introduction

Minimal invasive intervention is one of the most important themes in restorative dentistry. Total replacement of faulty restorations has been seen to be the most frequent procedure, and it is an important aspect of restorative dentistry in routine dental operations. 1 2 Repairing, refurbishing, or sealing damaged resin composites has been shown in several clinical studies to be a reliable alternative to total replacement, 3 4 5 effectively enhancing the longevity of the restorations. 6 7 In the case of secondary caries under resin composite restoration extended to the root or inadequate moisture control area, repair with resin-modified glass-ionomer cement (RMGIC) is another treatment option.

For the quality of the repaired restoration, a powerful adhesion between the aged resin composite and the RMGIC is needed. To explain the bond between aged resin composite and RMGIC, two strategies have been presented: (i) by penetrating the adhesive monomers into the aged resin composite-treated surface's irregularities, micromechanical retention is achieved and (ii) between resin composite and RMGIC, monomers are chemically bonded to the matrix or/and exposed filler molecules. The use of resin adhesive agents promotes RMGIC adherence to resin composites. 8 Furthermore, hydroxyethylmethacrylate integrated into the resin composite, builds a chemical adhesion with the RMGIC.

In the chemical adhesion of resin-based materials and silica-based materials, silane coupling agents play a significant role, and strong siloxane bonds were formed. 9 10 3-methacryloyloxypropyl trimethoxysilane is one of the most extensively used trialkoxysilanes in prosthodontics and restorative dentistry. 11 One of the most important steps in achieving good adhesion between resin-based materials and silica-based materials may be the use of silane. 12 13 Over the last decade, a new generation of universal adhesives has emerged. Some universal adhesives have silane coupling agent in the composition such as single bond universal (SU; 3M, Deutschland GmbH, Neuss, Germany), it may be improving bond strength between resin composite repaired with RMGIC. However, the shear bond strength (SBS) of chemical surface modification methods of aged resin composite repaired with RMGIC is underreported in the literature.

The purpose of this research was to explore the chemical surface modification strategies for resin composites that have been repaired using RMGIC. The work's null hypothesis was that the method of chemical surface modification of resin composite repaired with RMGIC does not differ.

## Materials and Methods

Table 1 shows the composition of silane coupling agent, adhesive, RMGIC, and resin composite used in this investigation.

**Table 1:** Materials used in the study

Material	Composition
RelyX ceramic primer (3M ESPE, Minnesota, USA) Lot: N988623	Ethanol, water, methacryloxypropyltrimethoxysilane
Single bond 2 (3M ESPE, Minnesota, USA) Lot: N378816	Bis-GMA, HEMA, DMA, methacrylate functional copolymer, filler, photoinitiators, ethanol, water
Single bond universal (3M, Neuss, Germany) Lot: 483316	10-MDP, Bis-GMA, HEMA, DMA, methacrylate functional copolymer, silane, filler, initiators, ethanol, water
RMGIC (Fuji II LC capsule, shade A2, GC Corporation, Tokyo, Japan) Lot: 1903191	Powder: Fluoroalumino silicate glass Liquid: UDMA, HEMA, polyacrylic acid, water, and camphorquinone
Resin composite (Filtex Z350 XT (A3D), 3M ESPE, Minnesota, USA) Lot: N994110	Silane treated ceramic, silane treated silica, silane treated zirconia, Bis-EMA-6, Bis-GMA, UDMA, PEGDMA and TEGDMA

Abbreviations: 10-MDP, 10-methacryloyloxydecyl dihydrogen phosphate; Bis-EMA-6, bisphenol A polyethylene glycol diether dimethacrylate; Bis-GMA, bisphenol A-glycidyl methacrylate; DMA, dimethacrylate; HEMA, 2-hydroxyethyl methacrylate; PEGDMA, polyethylene glycol dimethacrylate; TEGDMA, triethylene glycol dimethacrylate; UDMA, urethane dimethacrylate.

### Preparation of Bonding Specimens

Ninety resin composite rod specimens (Filtex Z350 XT [A3D], 3M ESPE, Minnesota, United States) were produced using clear silicone mold (the diameter is 5.0 mm and the thickness is 4.0 mm). The resin composite was loaded within the clear silicone mold, which was then light activated for 40 seconds (MiniLED, Acteon, Merignac Cedec, France) on both the top and bottom. Clear silicone mold was taken away from produced resin composite. The resin composite rods were thermocycled 5,000 rounds between 5 and 55°C, with a reside duration of 20 seconds and a movement time of 3 seconds each time. Acrylic was used to embed each resin composite rod in polyvinyl chloride tube. All sample surfaces were polished using silicon carbide paper 600 grid size (3M abrasive sheet, 3M, Minnesota, United States), after that 10 minutes ultrasonic cleaning with distilled water, and then 10 seconds of drying with triple syringe oil-free air.

### Chemical Surface Modification of Specimens

#### Phosphoric Acid Treatment

The specimen was treated with 37% phosphoric acid (Dentalife, Ringwood, Australia) for 15 seconds before being washed and dried with triple syringe oil-free air for 10 seconds.

#### Silane Coupling Agent Treatment

The specimen was treated with RelyX ceramic primer (RXP) (3M ESPE), and then 10 seconds of air-drying with a triple syringe.

#### Adhesive Treatment

The specimen was treated with single bond 2 adhesive (SB2) (3M ESPE) or SU adhesive (3M), and then 10 seconds of air-drying with a triple syringe, and after that light cured for 20 seconds.

The samples were sorted into 9 groups of 10 specimens each at random and surface modified using silane agent and/or bonding agent as follows: group 1, as a control, there is no surface treatment; group 2, etch + SB2; group 3, SB2; group 4, etch + RXP + SB2; group 5, RXP + SB2; group 6, etch + SU; group 7, SU; group 8, etch + RXP + SU; and group 9, RXP + SU.

A clear silicone mold (the diameter is 2.0 mm, and the thickness is 2.0 mm) was put on the top of specimen center, and after that injected with RMGIC (Fuji II LC capsule, shade A2, CG Corporation, Tokyo, Japan), and subsequently light cured for 40 seconds. All samples were kept at room temperature (25°C) until 30 minutes before being stored at 37°C in an incubator for 1 day within distilled water.

#### Shear Bond Strength Testing Procedure and Pattern of Fracture Inspection

A mechanical testing apparatus (AGS-X 500N, Shimadzu Corporation, Kyoto, Japan) was used to perform the SBS, at 0.5 mm per minute test speed. By dividing the bonding zone by the force at which the bond fractured, the SBS was obtained.

Under a stereomicroscope at ×40 magnification, the fracture characteristics of aged resin composites and RMGICs were analyzed. 12 13 14 The fracture modes were categorized into three groups: (1) an adhesive fracture (fracture on the interface between RMGIC and aged resin composite), (2) a cohesive fracture (fracture within RMGIC or aged resin composite), and (3) a mixed fracture (cohesive and adhesive failure in combination).

### Analysis of the Data

To examine the data, one-way analysis of variance was used, and Tukey's test was operated to establish significance level with a *p* -value of < 0.05.

## Result

The SBS mean values and standard deviations are shown in Table 2 . The greatest SBS values were showed in groups 4, 5, 8, and 9. Group 1 showed significantly lower SBS value.

**Table 2:** The mean SBS, standard deviation, and failure mode percentage

Group	Mean SBS ± SD		Failure mode	
		Adhesive	Mixed	Cohesive
1. No treatment	8.14 ± 2.36 ^a^	100	0	0
2. Etch + SB2	17.25 ± 2.04 ^b^	80	20	0
3. SB2	16.79 ± 3.41 ^b^	80	20	0
4. Etch + RXP + SB2	24.65 ± 2.89 ^c^	60	40	0
5. RXP + SB2	24.02 ± 1.94 ^c^	70	30	0
6. Etch + SU	18.32 ± 2.49 ^b^	80	20	0
7. SU	17.58 ± 2.55 ^b^	90	10	0
8. Etch + RXP + SU	25.36 ± 1.58 ^c^	70	30	0
9. RXP + SU	24.18 ± 3.11 ^c^	60	40	0

Abbreviations: RXP, RelyX ceramic primer; SB2, single bond 2; SBS, shear bond strength; SD, standard deviation; SU, single bond universal.

Note: The value with identical letters indicates no statistically significant difference.

Table 2 summarizes the distribution of failure mode after a SBS test. After fracture, all samples in group 1 were categorized as adhesive failure. Groups 2 to 9 found mixed failures between 10 and 40%, and adhesive failures between 60 and 90%.

## Discussion

This study examined the chemical surface modification methods of resin composite repaired with RMGIC. The results demonstrate that the SBS of each group is significantly different. As a result, the null hypothesis was disproved.

For clinical effectiveness, the old resin composite and RMGIC must adhere to each other in a durable and reliable approach. To improve mechanical retention, the old resin composite's surface roughness must be increased and produce chemical adhesion between the resin composite and RMGIC, also use an appropriate adhesive agent for surface wetting that is strong enough. Furthermore, there have been several attempts to develop primers that are specifically designed for resin composites. Nevertheless, bonding the RMGIC to the old resin composite is still a challenge. To enhance the repair bond ability of resin composites, several micromechanical surface preparation procedures and chemical surface treatment by adhesive systems have been recommended. Mechanical roughening using burs can be used to provide surface roughness on old resin composite restorations. Brosh et al 15 reported that the maximum bond strength is succeeded by using a diamond bur or sandblasting on the surface. In this research, SBS between old resin composite and RMGIC was unaffected by 37% phosphoric acid; consequently, bond strength was not improved. Loomans et al 16 found that surface roughness in resin composites was unaffected by phosphoric acid. Moreover, Bahari et al 17 reported that rough and regular surfaces were significantly smoothed after etching with 37% phosphoric acid, surface roughness has decreased, as a result, the bond strength was significantly reduced.

When adhesives are used following surface pretreatments, the bond ability of the repair restoration is considerably increased. 18 19 The utilization of a bonding resin on an old resin composite improves the surface's wettability by penetrating and polymerizing into the prepared surface, resulting in micromechanical retention. 20 The adhesive resin could infiltrate within the surface roughness and reinforce them to create resin tags for adhesive. Nevertheless, Irmak et al 19 found that one-step adhesives are more hydrophilic and contain more acidic monomers than two-step adhesives. Due to the lack of a separate hydrophobic adhesive layer, their ability to bond to resin composites may be impaired. In terms of preventing microleakage, Celik et al 21 said that one-step bonding resins might not be the best choice for resin composite repair. In this research, shear bond ability between aged resin composite and RMGIC was affected by application of adhesive resin prior to being repaired with RMGIC, consequently, SBS was enhanced. The application of the adhesive material is indeed crucial in RMGIC repairs of old resin composites.

To use a silane agent enhances the wettability of the repaired surface by encouraging chemical bonding between the silica or glass fillers and the resin matrix material, according to several studies. 12 13 22 23 24 25 Staxrud and Dahl 26 reported that the repair of resin composites with a universal adhesive proved as successful as a pure silane prior to the use of an adhesive agent. Moreover, Fornazari et al 27 found that the application of a universal bond containing silane eliminates the requirement for a separate use of silane coupling agent in the clinical procedure for aged resin composite repair. Conversely, Silva et al 28 reported that the prior application of silane was still necessary for aged resin composite repair. Kalavacharla et al 29 indicated that although SU adhesive incorporates silane agent, it does not enhance bond strength as much as pure silane agent. The pH of SU is around 2.7. 30 The silanol parts in silane agent may experience early self-condensation reactions because SU is acidic through the acidic monomer, 10-methacryloyloxydecyl dihydrogen phosphate. 31 Nevertheless, the silane quantity in its formulation is unknown, and it may be inadequate to improve repair bond strength. This research found that the aged resin composite repair bond strength using RMGIC still recommended the silane coupling agent's application prior bonding agent for best results. The silane coupling agent's application enhances the wettability of the repaired resin composite surface by advocating chemical bonding between the silica or glass fillers and the resin matrix in resin composite and RMGIC.

As for the failure mode, most of the failures in the investigation were adhesive failures in all the experimental groups. High SBS was commonly associated with mixed failure, as showed from groups 4, 5, 8, and 9. The SBS was significantly higher when mixed with silane coupling agent paired with the adhesive repair method.

## Conclusion

Under the scope of this study's conditions, the use of bonding agents enhances the resin composite's wettability and allows it to bond to RMGIC, thus encouraging strong shear bond ability between resin composite and the RMGIC. Furthermore, the silane coupling agent's application prior bonding agent showed significantly higher shear bond ability between resin composite and the RMGIC.
